# Compassionate engagement of communities in support of palliative and end-of-life care: challenges in post-pandemic era

**DOI:** 10.3389/fmed.2024.1489299

**Published:** 2024-10-18

**Authors:** Joel Vieira Vitorino, Beatriz Veiga Duarte, Amira Mohammed Ali, Carlos Laranjeira

**Affiliations:** ^1^School of Health Sciences, Polytechnic University of Leiria, Leiria, Portugal; ^2^Palliative Care Unit, Portuguese Institute of Oncology of Coimbra, Coimbra, Portugal; ^3^Department of Psychiatric Nursing and Mental Health, Faculty of Nursing, Alexandria University, Alexandria, Egypt; ^4^Centre for Innovative Care and Health Technology (ciTechCare), Polytechnic University of Leiria, Leiria, Portugal; ^5^Comprehensive Health Research Centre (CHRC), University of Évora, Évora, Portugal

**Keywords:** compassionate communities, death literacy, palliative care, post-pandemic era, advance care planning, psychosocial support, end of life care, telehealth

## Abstract

Over the years, humanity has faced various global crises of different kinds that have caused great suffering in the community, such as wars, slavery, torture or the Holocaust, but also climate change, economic crises, or sanitary disasters. The recent pandemic posed a barrier to palliative and End-of-Life (EoL) care, as the need for physical distance made it difficult to retain essential human interactions while minimizing the risk of viral transmission. During the COVID-19 pandemic, the robustness of supportive networks (i.e., family, friends, neighbors, and community members) determined whether someone experienced a calm death at home or an unnecessary hospital admission, labeled as an ‘emergency’. In this vein, active establishment and strengthening of such networks are the foundation of compassionate community efforts. Firstly, providing both physical and emotional support to the entire network of caregivers enhances their ability to care for others and improves the overall experience of death, including the process of dying and the ensuing bereavement period. Furthermore, individuals can enhance their own physical and mental health by practicing compassion. The ability of networks to withstand and recover from physical and emotional challenges, while maintaining strong and supportive relationships among its members, depends on the health and overall well-being of those members. Therefore, we argue that active community participation and death education can strengthen a community’s capacity to assist people facing death, dying, and bereavement.

## 1 Introduction

Promoting the compassionate engagement of communities and advocating for death literacy are essential components in organizing populations for potential global crises. The COVID-19 pandemic highlighted how people and communities are unprepared for death, i.e., unable to deal with the psychological, emotional or spiritual issues associated with the process of death and dying. According to a recent concept analysis about death unpreparedness due to COVID-19 (1), three main attributes stand out: (1) individual attributes, including aspects related to the paucity of death and grief literacy; (2) relational attributes, in particular the issue of dying unaccompanied; and (3) contextual attributes, in particular the disruption of compassionate care and collective mourning and grieving. Death unpreparedness increases death-related anxiety and stress, conditions a positive awareness of death, increases bereavement overload and limits people’s capacity to appreciate their lives (1). Therefore, it is crucial to integrate compassionate community networks with work social services and natural support systems to promote a social approach to EoL care (2, 3).

Like health, as defined by the World Health Organization (WHO), death, loss, and care are also everyone’s duties. The Compassionate Cities movement, promoted by the Public Health Palliative Care International (PHPCI) Organization, advocates that compassionate communities are “those that publicly recognize people at the end of life and their needs, and are conscious of seeking and involving all major sectors of the city to help through care and accompaniment to reduce the social, psychological and health impact of difficult life processes and situations, especially those related to disability, aging, dependency, end of life, caregiver burden, grief and bereavement” (4). Likewise, considering the agenda for Sustainable Development Goals (5) and the WHO’s guidelines for a community empowerment strategy (6), an approach based on creating supportive environments based on people’s needs is fundamental, while attending to the needs of future generations and reorienting health services through a relationship of collective responsibility shared by individuals, community groups, health professionals, health services and government.

The purpose of this paper is to discuss how a compassionate engagement approach can promote and support the development of a common understanding of death, dying and bereavement. Besides, death literacy emerges from the need to understand how communities come together in care, especially in equipping people to cope with loss, grief, and the concept of mortality (i.e., the people’s awareness and cognitive recognition of their death as an unavoidable event). Lastly, this paper highlights some implications for the future of palliative and EoL care delivery in a post-COVID-19 world.

## 2 Compassionate communities: an essential transition in the post-pandemic era

Compassionate communities emerged from an international movement seeking to implement a public health strategy to EoL care, based on the empathetic assumption of shared social responsibility in the face of inevitable suffering rather than the exclusive institutionalization of responses (7–9). Aspects such as health, illness, death and loss are understood as natural aspects of human existence. This approach promotes a holistic outlook of the person, focusing on collective and individual well-being in its different dimensions: biological, psychological, spiritual and social.

Compassionate communities promote social capital’s potential by fostering and encouraging relationships of mutuality through the empowerment of citizens who are expected to play an active role in their communities. These networks of support also contributing to the creation of meaningful social connections capable of increasing individual support systems while encouraging the development of communities capable of withstanding adversity (i.e., resilient communities) (10). This community movement includes various social actors, from formal agents (health and social care networks) to informal agents, who belong to different contexts: workplaces, community centers, cultural and religious organizations, and recreational and sports associations, among other organizations with different levels of structural formality (3, 10, 11). Different levels of development derive from different political paradigms, but also from different levels of the population’s literacy, without neglecting the context and culture associated with attitudes toward the EoL (12). Considering the advancement of health sciences and medicine, the perspective of treating illness as having a successful outcome has been consolidated in modern societies, thereby sometimes overlooking the inevitability of death and neglecting the complexity of the response to EoL adjustment processes (13).

Over the last decade, there has been a growing recognition that communities have been marginalized and EoL care has been increasingly institutionalized. Currently, scholars analyse institutionalization as the process of relocating older, ill, and dying people to care facilities and hospitals (14). This practice involves removing dying and death from regular daily life and is linked to the societal stigma surrounding old age, disease, dying, and death. The discourse examines the spatial distribution of dying in various locations, which are significant not only due to their physical characteristics but also because of the interconnected social environments they offer for the process of dying. The quality of a person’s death is influenced not just by the location, but also by their specific social support structure. However, certain areas or situations are closely linked to either positive or negative practices of care and support. The familiar environment and the intimacy, tranquility, and steadiness provided by home care make it a suitable social context for dying, as well as for preserving ontological security and relational connection (15). Potential homes are not limited to private households but could include nursing homes that provide suitable circumstances for their residents. Conversely, societal discussions associate hospitals with onerous and potentially superfluous treatment as a component of advanced medical technology, impatience, noise, and a dearth of privacy. Hospitalization is discursively presented as antithetical to a good death (16).

In contrast, compassionate communities make use of social connections, reciprocity and trust, providing a paradigm shift in palliative care policy and practice. This shift has led to the development of new practical initiatives promoting EoL activities and models of care that are delivered within a community framework, promoting personal and community empowerment (17). Such training increases the ability to develop and maintain knowledge about EoL care in the community and is a catalyst for using support systems to solve problems, make decisions, communicate and act more effectively when someone in the community is dying (11). Specific examples of compassionate community interventions include (a) providing training for health professionals, volunteers, caregivers, and faith communities to facilitate discussions on death and dying; (b) promoting TV and radio coverage that encourages the choice to die at home; (c) organizing “cultural” events, such as art exhibitions, death cafés, and remembrance festivals; (d) establishing community group sessions in various settings such as senior housing, churches, assisted living facilities, and businesses; and (e) advocating for research organizations to prioritize EoL research, including community-based participatory studies (3, 18).

Notwithstanding, several barriers influence the involvement of communities, such as inadequate preparation due to lack of competence; the voluntary nature of programs; cultural, social and economic differences and the nature of vulnerabilities; time constraints and conflicts with individual priorities; and conflicts of interest (3, 13, 19). To mitigate these barriers, it is important to identify the main individual and social gains from the development and implementation of compassionate communities, including significant improvements in social relationships, the ability to cope with daily activities, and increased support networks. Formal services and networks are less effective in these aspects, especially in environments of greater social isolation (20). Realities are disparate when it comes to developing compassionate communities, and the emerging challenges certainly include raising awareness of compassion-based care among citizens, emphasizing social reinforcement, the helping relationship, and the ability of citizens to actively participate in the development of palliative care in their communities (21).

## 3 Death literacy: empower both the self and the other

Death literacy, emerging from the need to understand how communities come together in care (17), is understood as “the knowledge and skills necessary to enable access to, compression of and informed choices about end-of-life and dying care options” (17). This concept requires systemic and behavioral change, based on components such as skill, knowledge, experiential learning, and community action (22). Additionally, death literacy plays a critical role in equipping people to cope with loss, grief, and the concept of mortality. Present-day society often shies away from openly discussing death, which can lead to emotional and spiritual unreadiness when facing crises. By advocating for death literacy, we empower individuals to address questions of human existence more adeptly and mindfully, fostering a healthier approach to these inevitable aspects of life. Death literacy has a significant impact on reducing anxiety about death (23) and promotes individual awareness regarding decision-making on fundamental issues throughout life, especially at the EoL, fostering greater involvement in the definition of an advanced care plan (24). Interestingly, research suggests that responses to awareness of mortality vary between younger and older individuals. Developmentally, younger people typically focus on current worries and future ambitions, whereas older adults tend to prioritize deeper life meanings, which makes them more attuned to the broader implications of mortality (25). Hence, the progression of development implies that coping mechanisms for the fear of death change with time.

Several factors influence the existence of death literacy. They can be organized into three groups: sociodemographic factors (age, gender, level of education, spiritual and religious beliefs); professional factors (health professionals show better indicators of death literacy); and factors related to experiences of support and care in situations related to death and the EoL (in a personal, professional or volunteer context) (26). It is also important to consider moral differences and cultural diversity as relevant aspects in the different conceptual assumptions related to death and dying (27).

Death literacy can be seen as a health resource or outcome, insofar as it mediates better health indicators—if we consider the benefits of being able to deal with death and the EoL—or, alternatively, a result of individual and/or collective experiences of dealing with these processes (28). It is important to move beyond “talking about death” to an in-depth conversation that develops criticism and awareness, enabling social commitment that translates into community empowerment and, above all, active care and creating compassionate communities (22). As a community and social need, death literacy can involve formal agents or concerted actions in certain target groups, such as health professionals or students, which translates into the strengthening and development of higher levels of competence in EoL issues (29).

In recent decades, Western culture has systematically suppressed reflection about death and dying. This resulted in individuals ill-equipped to handle the overwhelming amount of news related to mortality during the pandemic time (30). Recently, certain educational programs were created to assist individuals in becoming more comfortable with the concept of death. These programs encourage open discussions about death among peers and provide opportunities for individuals to express their thoughts and emotions through artistic means. This creative approach allows individuals to explore the concept of death and dying in a more unrestrained manner (31). Interventions to promote death literacy are diverse, ranging from innate development through experiential learning about EoL situations and the death of someone significant (32, 33); classic approaches to skills development through formal education programs in a face-to-face training methodology and using pedagogical strategies such as roleplay (34) or an e-learning training methodology (29); professional interventions in health care contexts (35); but also through more or less formal community interventions, such as death cafés (36), or games using “DöBra cards”—a tool to support conversations about values and preferences for future care at the EoL (37). Another effective methodology utilized in death education is photovoice. In this approach, participants are instructed to create photographs that explore the topic of death and dying, capturing their perceptions and representations. These photographs are then shared with the group and serve as a basis for discussion and reflection (31, 38). These experiences have shown that actively addressing the topic of death and the accompanying unpleasant emotions can effectively enhance individual resilience and reduce fears (39). Death education seeks to facilitate open discussion and reflection on topics that are typically avoided in everyday conversations due to their tendency to evoke feelings of concern and sadness (40). Increased awareness of the fear of death and the subsequent protective mechanisms helps individuals confront challenges that occur from mortal circumstances and enable them to empathize with those experiencing personal loss (36, 37). Furthermore, studies have demonstrated that contemplating mortality and the fragility of human existence can also alleviate anxiety and enhance the ability to handle health-related information well (41).

## 4 Implications for the future of palliative and EoL care delivery

The impact of major crises has a knock-on effect on the organization of societies, with significant consequences for the health of their populations (42–47), requiring health systems to organize themselves to promote people’s health, integrating the community as an active and central element in the design and implementation of a public health strategy for palliative care (48). Clearly community empowerment, which allows people to gain control over decisions and actions that affect their lives (49). That is a path of partnership with institutional health services that can bring people’s real needs to the forefront, including them as active agents and key elements in defining solutions that respond to their problems.

Thus, it is important to develop responses addressing the transversal suffering caused by the foreseeable increase in chronic diseases (50), and to ensure a comprehensive and consistent response to possible new crises, namely in three areas: telehealth palliative care services, psychosocial support and advance care planning.

### 4.1 Telehealth palliative care services

The COVID-19 pandemic demanded swift adaptations in palliative care delivery by healthcare systems to address the evolving needs of patients and their caregivers. Telehealth is an essential tool for expanding the reach and availability of palliative care. It is particularly important for patients residing in remote and rural areas (51, 52) or for those without financial resources to access the required technologies. These individuals need to have guaranteed access to expert treatment. The most effective telehealth practices involve establishing rapport, setting the agenda, responding empathically to emotions, conveying information, and effectively concluding the visit to ensure a user-centered design (53, 54). Nevertheless, additional comprehensive testing is required in real-world settings. Promising areas for further investigation involve the evaluation of algorithms to determine which patients would derive advantages from telehealth, the examination of technological proficiency, the assessment of communication quality in telehealth interactions, the testing of telehealth effectiveness in diverse settings (such as cultural or clinical contexts), and the exploration of perceptions of the quality of care by both patients and caregivers (53, 55, 56).

### 4.2 Psychosocial support

The pandemic highlighted the challenge of maintaining consistent care, particularly in the fields of palliative care (57). It allowed healthcare teams to fully recognize the importance of comprehensive support services (including social, psychological, and spiritual assistance) that enhance the quality of experiences for both patients and caregivers. These services became more difficult to access or were no longer available during the pandemic. Furthermore, evidence suggests that patients and their caregivers frequently face difficulties in physically accessing support services located in palliative centers due to transportation or scheduling constraints. As a result, they tend to choose community-based support that is closer to their homes (58). With the shift toward outpatient EoL care, establishing stronger ties between Healthcare Professionals (HCP) and the community is crucial. This includes implementing efficient procedures to connect patients and caregivers with community partners outside of clinic visits. Previous research has provided evidence that nurse-led transitional care models are effective in enhancing the consistency of care for older persons with chronic diseases when they move between different healthcare settings (59). Palliative care nurses may benefit from utilizing modified transitional care models to enhance the provision of comprehensive and continuous community-based supportive services across the entire trajectory of care (60). Implementing proactive strategies to support palliative needs within basic palliative care can potentially alleviate the strain on already overwhelmed speciality palliative care programs.

The scarcity of psychological support services for caregivers, particularly during the period of bereavement, as well as for HCP, became evident throughout the pandemic. Palliative care programs should establish more structured bereavement programs, including innovative approaches like internet and mobile-based interventions to effectively expand the reach of grief services (61).

While HCP have long been familiar with this issue, the pandemic has heightened the awareness of both healthcare institutions and the general public regarding the moral and psychological suffering experienced by HCP, and the subsequent impact on their mental health. A significant number of clinicians, particularly nurses, experienced mental health consequences to the point that they seriously considered leaving the healthcare field (62–64). However, the provision of comprehensive mental health assistance for HCP is still far from desirable levels. The widespread disparity requires healthcare systems to strengthen and enhance the mental health support services provided during the pandemic. These services include monitoring psychological distress, offering in-person and/or virtual psychotherapy, providing education on resilience and mindfulness, and offering tools for debriefing and stress management (65, 66). These interventions should be accompanied by comprehensive investments and policies at the system level to safeguard the nursing workforce and emphasize their long-term sustainability even after the pandemic’s effects have subsided (66, 67).

### 4.3 Advance care planning

Kellehear stated that incorporating ideas of community participation and development is a key component of the overall strategy to promote the utilization of Advance Care Planning [ACP] (11, 68). Expanding the principles of promoting health and well-being and reducing harm can provide a more comprehensive knowledge of how the usage of ACP can be further improved to better serve professionals, patients, and families. Within the framework of ACP, the promotion of health and well-being emphasizes the creation of supportive settings, not only to address the demands of care but also to foster positive connections and goals for both patients and caregiving networks. Kelley (2) explains the functioning of these networks and how they can be utilized to improve the significance and worth within the caring network, while also managing the challenges it faces.

ACP discussions are crucial to guarantee person-centered care, particularly in times of crisis. The rise in the number patients with COVID-19 requiring intensive care in hospitals complicated the delivery of medical care to other critically ill patients, leading to some patients not receiving the necessary medical attention. Healthcare facilities struggled to treat the overwhelming number of COVID-19 patients and the other seriously ill patients, hindering discussions of ACP. Furthermore, places in the hospital that ensured social distancing during the ACP discussion and signing the ACP face-to-face were lacking (69). Healthcare professionals faced difficulties in effectively addressing the beliefs and wishes of patients regarding end-of-life issues, formulating care strategies that aligned with these wishes, and providing individualized care. The typical bereavement process was hindered, placing a psychological burden on patients, families, and healthcare workers, as a result of ethical dilemmas like triage and resource management during the COVID-19 pandemic.

Research indicates that mass media and online ACP resources are essential in promoting community-based ACP (69). A vast amount of information about the COVID-19 pandemic was disseminated by mass media worldwide. For instance, an innovative and user-friendly ACP website (PREPARE) was created in the United States (70). Its purpose is to educate individuals on identifying their life priorities and desired lifestyle, effectively communicating their preferences to family and doctors, and making informed medical decisions that align with their personal values and beliefs. This website is interactive, easily accessible, and completely free of charge. Likewise, incorporating crucial ACP data into the Electronic Health Record is imperative to enable multiple clinicians, from disparate healthcare organizations, to access and understand the information that holds the utmost significance to patients. This facilitates the clarification of patients’ preferences for EoL care and allows regular review of all documents to ensure they continue to accurately reflect their wishes (71). Besides, HCP need to assert and utilize their position in facilitating ACP throughout the entire trajectory process. In this vein, the pandemic underscored the need to provide ACP education to hospital and home care workers, implementing a telemedicine-based system for immediate dissemination of ACP information among hospitals and facilities, and facilitating debates about ACP within the community (72, 73). Patients and caregivers may now be more open to these discussions due to heightened social awareness regarding their significance brought about by the pandemic.

## 5 Final remarks

In a changing world, the prevailing state of isolation and self-centeredness necessitates a re-evaluation of the concepts and methods of healthy communities by incorporating compassion. This entails reestablishing connections via genuine acts of care, actively engaging with others, and fostering supportive networks in caregiving. This movement integrates the experiences of death and loss into our health frameworks and infuses the notion of compassion into our health policies (10). The primary goal is to raise awareness within the community and encourage their active participation in end-of-life care. The COVID-19 pandemic has intensified the issue of communal solidarity and conviviality, and promoted Compassionate Community interventions due to the increased attention given to the experiences and support requirements of the bereaved and those nearing the EoL.

## Data Availability

The original contributions presented in the study are included in the article/supplementary material, further inquiries can be directed to the corresponding author.
